# Distinct mechanisms of temporal binding in generalized and cross-modal flash-lag effects

**DOI:** 10.1038/s41598-019-40370-7

**Published:** 2019-03-07

**Authors:** Ryusuke Hayashi, Ikuya Murakami

**Affiliations:** 1 Human Informatics Research Institute, National Institute of Advanced Industrial Science and Technology (AIST), Umezono 1-1-1, Tsukuba, 305-8568 Japan; 2 Department of Psychology, The University of Tokyo, 7-3-1 Hongo, Bunkyo-ku, Tokyo Japan

## Abstract

It remains unknown how the brain temporally binds sensory data across different modalities and attributes to create coherent perceptual experiences. To address this question, we measured what we see at the time we experience an event using a generalized version of the flash-lag effect (FLE) for combinations of visual attribute (bar orientation, face orientation, or face identity) and probe modality (visual or auditory). We asked participants to judge the content of rapidly and serially presented images seen at the same time as a briefly presented visual (flash) or auditory (click) probe and estimated the “time windows” contributing to decisions using reverse correlation analysis. We also used displays in which the visual attribute of a stimulus continuously changed and measured FLEs around abrupt flip in change direction and at the initiation and termination of a sequence. We consistently found clear latency-difference effects, which depended on visual attribute for the visual probe but did not for the auditory probe. The intra-modal FLE can be explained in terms of differential latency and temporal integration, but the cross-modal FLE is suggested to operate via a distinct mechanism; the content of a successive visual stream experienced after the awareness of a click is interpreted as simultaneous with the click.

## Introduction

Our perceptual system is confronted with the difficult task of estimating how the external world develops in time. To accomplish this, the system employs a multitude of sensory signals provided by multiple sensory modalities such as vision and audition. These are initially processed in separate pathways, within each of which multiple attributes of stimuli are extracted via subdivided mechanisms in the computational hierarchy. For example, visual processing at the cortical level begins in area V1 (Hubel & Wiesel^1^) and then proceeds along both ventral and dorsal pathways (*Ungerleider* and Mishkin^2^; Goodale and Milner^3^), gaining complexity at progressively higher cortical levels. Thereafter, the brain somehow binds different modalities and attributes to represent a world of coherent perceptual entities (Treisman & Gelade^4^, Fujisaki and Nishida^5^). Exploring what we see in terms of visual attributes at the time we experience an event is important when seeking to elucidate the cross-attribute/modality-binding problem.

A flashed stimulus typically appears to lag behind a continuously moving stimulus even if they are aligned in space and time; the phenomenon is termed the “flash-lag effect (FLE)” (MacKay^6^; Nijhawan^7^). The FLE does not refer only to moving stimuli; it is generalizable to other visual attributes such as changes in color, spatial frequency, etc., when the attribute changes smoothly at a fixed location (Sheth *et al*.^8^). Moreover, the FLE can occur cross-modally, e.g., when a visual motion is compared with an abrupt onset of sound that serves as an auditory equivalent of a visual flash (Alais *et al*.^9^; Hine *et al*.^10^). As the FLE has been found over a wide range of attributes/modalities, it is a useful psychophysical tool when investigating the mechanism of temporal binding among various types of information at the time of an abrupt event, either visual or auditory, hereinafter termed a “probe.”

As neurons respond faster to some stimuli than others, neural latency may influence the FLE (Krekelberg & Lappe^11^). However, it remains unclear how visual information is temporally integrated to construct the percept of a visual attribute at visual probe onset (intra-modal binding) or at auditory probe onset (cross-modal binding) despite differences in latency for processing.

In this study, we focused on the following four points. First, we sought to quantify the temporal integration of visual information for several stimulus attributes. To this end, we used a rapid sequential visual presentation (RSVP) (Murakami^12^; Murai & Murakami^13^) and applied reverse correlation analysis (Simoncelli *et al*.^14^; Ringach & Shapley^15^; David *et al*.^16^; Hayashi *et al*.^17^) to estimate which display frames in the RSVP contributed to the judgment of visual content being simultaneous with the probe. The decision regarding what was perceived at probe onset was assumed to be based on a weighted sum of a visual sequence around the probe. Reverse correlation analysis estimates the linear component (the first-order kernel) of this weight function by summing a randomly presented visual sequence conditional on an observer’s binary responses. We refer to this temporal weight function as the “time window.” Second, we reasoned that if latency affected the time window, the effect would be maximal if we used visual attributes with diverse latencies. Orientation is first encoded in V1 neurons (Hubel & Wiesel^1^) but face is explicitly represented only at the stage of the inferior temporal (IT) cortex (Desimone *et al*.^18^). Moreover, posterior IT is selective for face orientation regardless of face identity, whereas anterior IT is selective for face identity rather than orientation (Freiwald and Tsao^19^). Therefore, we measured time windows using face-orientation and face-identification judgments, as well as bar-orientation judgment. Third, we also used continuously changing visual stimuli to see whether the time window estimated via RSVP reflects a general property of the binding mechanism and also explains perception in other situations in which visual attributes change smoothly and continuously, as in conventional FLE studies. Fourth, the generalized FLE has never been studied cross-modally; although a cross-modal FLE between an abrupt sound and visual motion has been reported (Arrighi *et al*. ^20^), visual attributes other than motion have never been tested. We examined differences between intra-modal and cross-modal FLEs using a visual flash and an auditory click.

## Experiment 1

We used the RSVP and reverse correlation analysis to estimate time windows. We chose three different visual tasks requiring processing at different cortical stages and used visual and auditory probes to explore differences between the intra- and cross-modal binding mechanisms.

## Methods

### Observers

Author RH and naïve observers with normal or corrected-to-normal vision participated (N = 16). All experiments were performed in accordance with the principles embodied in the Declaration of Helsinki and were approved by the institutional review board of the National Institute of Advanced Industrial Science and Technology (AIST). Written informed consent was obtained from all participants prior to the start of the experiments, after providing each participant with a written explanation of the aim and scope of the research.

### Procedure

**Figure 1:**
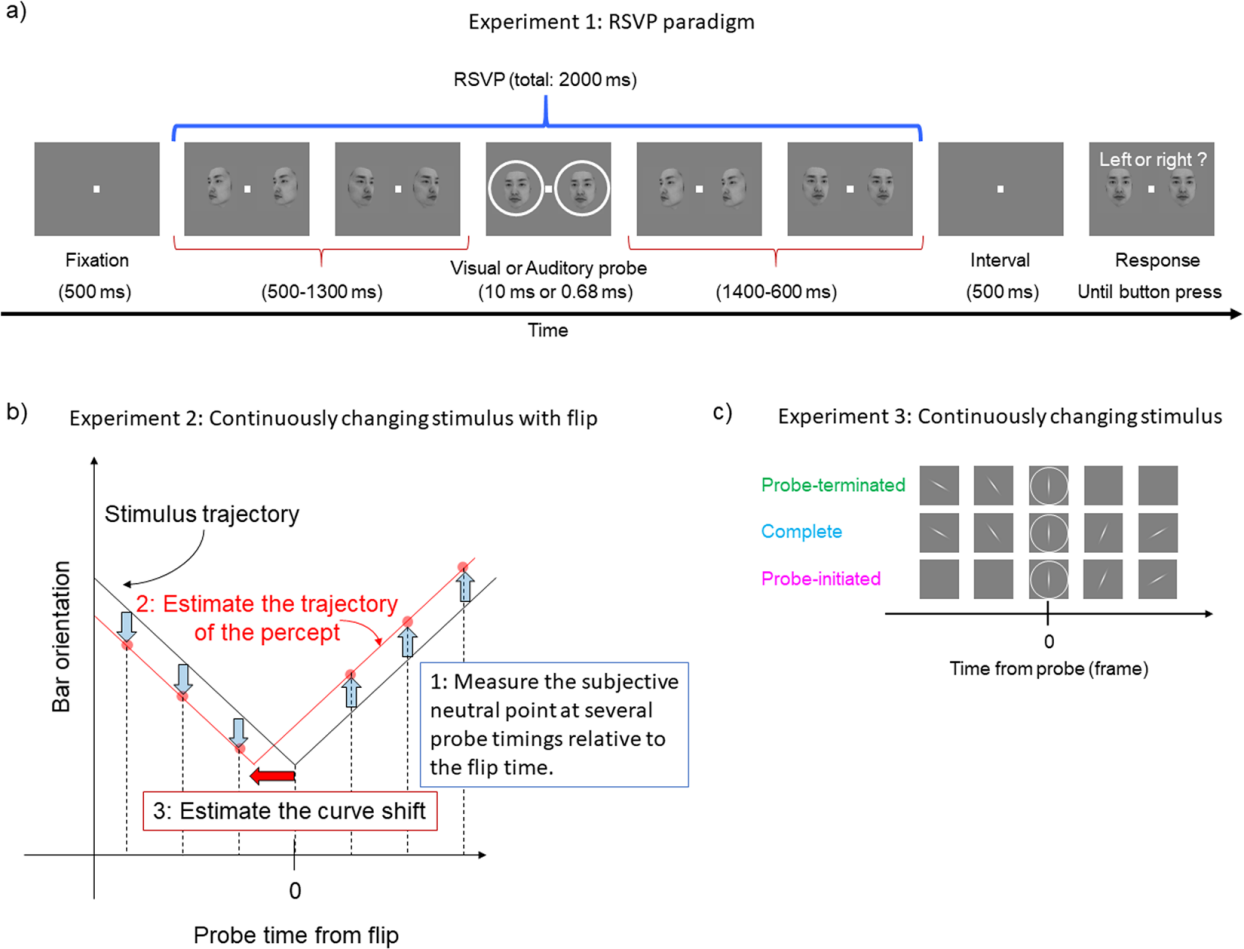
(**a**) Schema of the procedure of Experiment 1. (**b**) Schema of the procedure of Experiment 2. (**c**) Examples of visual sequences under the “probe-terminated,” “complete,” and “probe-initiated” cycle conditions in Experiment 3.

Visual stimuli were displayed on a CRT monitor (Mitsubishi Electric, RDF19S; refresh rate 100 Hz) under the control of Psychtoolbox-3 Extensions software (Kleiner *et al*.^21^) running in the Matlab (Mathworks) programming environment. At the beginning of each trial (Fig. 1a), a white solid square was displayed as a fixation point at the center of a uniform gray screen (37.8 cd/m^2^). After a 500-ms fixation period, randomly chosen images (20 of 151 different images) were sequentially presented every 10 frames (100 ms). A visual flash (a white ring surrounding each image, one frame long) or an auditory click (a single pulse, 0.68 ms long, synchronized with screen refresh and transmitted to headphones, Hitachi Maxell Ltd., Vraison VH-OH48) served as the probe and was delivered randomly during RSVP. After a 500-ms blank interval, each participant reported the content of the image seen at probe onset by pressing one of two buttons. The button-press triggered the next trial. Each stimulus was confined within 9 × 9 deg. Identical images were displayed at 4.5-deg eccentricity to the left and right of the fixation point to render it easier for the observers to gaze at the fixation point throughout the RSVP. Each session comprised 100 trials and there were four sessions for each condition.

### Visual Stimuli and Tasks

In each session, one of the following three tasks was chosen in pseudo-random order (see Supplementary Information and Fig. S1 for details):

- **Bar orientation task:** The visual stimuli were white bars, the orientations of which were within ± 60 deg from the vertical in 151 steps. We assigned stimulus indices ranging from –75 to + 75 to these images, depending on the bar orientation. The task was to report whether the bar seen at probe onset tilted counterclockwise (CCW) or clockwise (CW) from vertical.
- **Face orientation task:** The visual stimuli were monochromatic facial images in which the head orientation was within ± 60 deg in azimuth from the front in 151 steps. We assigned stimulus indices ranging from –75 to + 75 to these images, depending on the angle of face orientation. The task was to report whether the face seen at probe onset faced left or right.
- **Face identity task:** The visual stimuli were monochromatic front facial images morphed from persons A to B (both were Asian males easily distinguished from each other by our observers) in 151 steps. We assigned stimulus indices ranging from –75 to + 75 depending on the morphing level. The task was to report whether the face seen at probe onset looked more like person A’s or B’s. The same two persons (A and B) were used throughout the study. All facial images were matched in terms of image histograms to minimize difference in low-level features such as luminance and contrast.

### Data analysis

We used reverse correlation analysis to estimate the extent to which observers relied on images presented at various times when reporting what they saw at probe onset. Note that although the images were refreshed every 10 frames, the probe was delivered randomly, with a resolution of only one frame, irrespective of the phase of image duration. Thus, each time window was calculated with a resolution of one frame (10 ms). We also estimated a two-dimensional window, or a weight map on time-intensity domain, using the joint weights of timing and stimulus intensity for perceptual decision-making. For details about estimation of time windows and for the limitation of reverse correlation analysis in relation to the non-linear effect, see Line 75–97 in Supplementary Information.

## Results

### Visual probe

**Figure 2:**
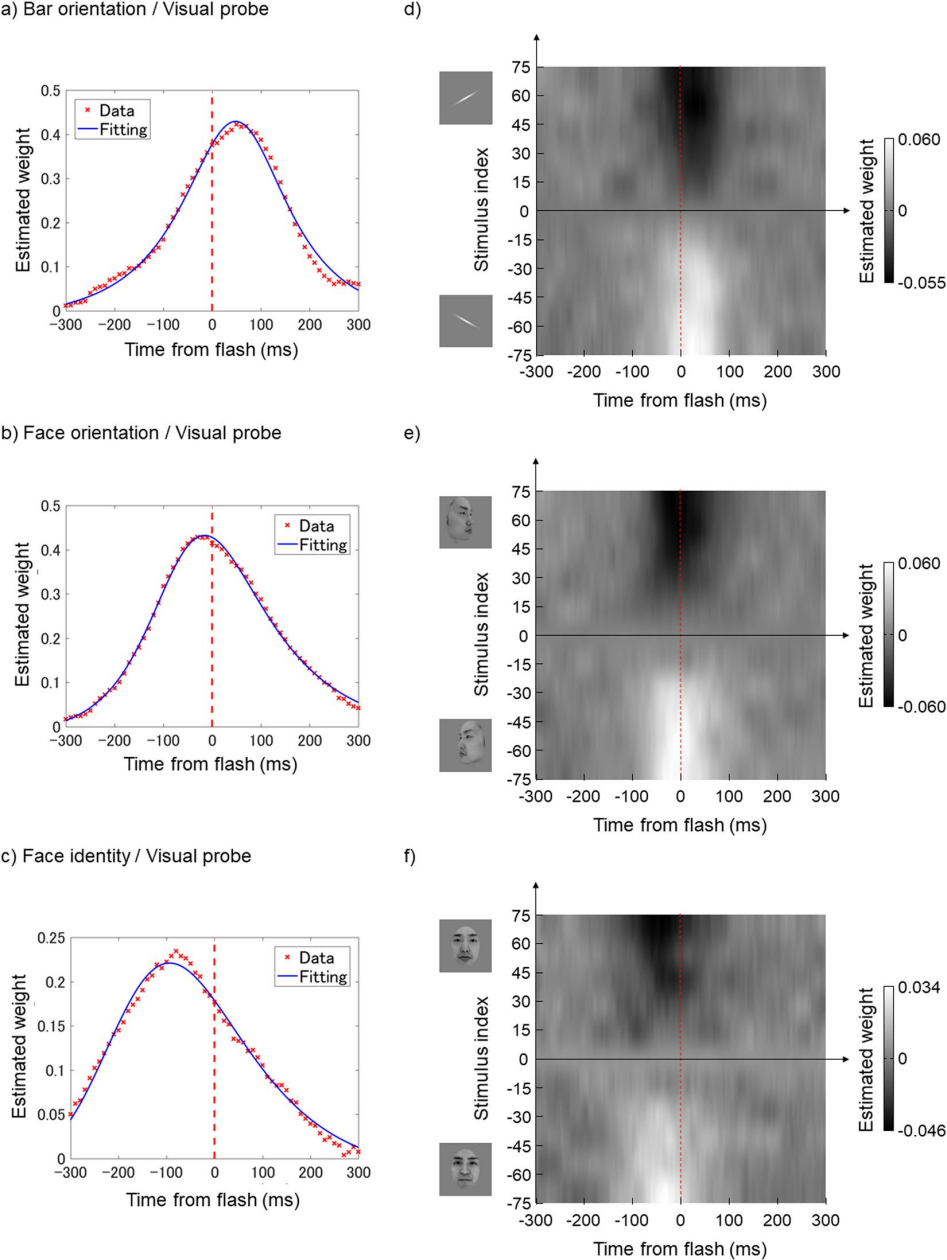
The results of Experiment 1 (flash stimulus / visual probe). The red broken line denotes flash onset. Left column: The time window for the flash, estimated using data averaged across all observers; (**a**) Bar orientation task, (**b**) Face orientation task, and (**c**) Face identity task. The red crosses indicate the weight estimated for each frame and the blue curves indicate the best-fit curves using the non-central t-distribution. Right column: the weight map for the flash condition in the time-intensity domain, estimated from data averaged across all observers; (**d**) Bar orientation task, (**e**) Face orientation task, and (**f**) Face identity task. Brighter pixels indicate how much the image at the corresponding time and stimulus intensity contributed to reporting that “the bar was tilted CCW” (bar orientation task), “the face was facing left” (face orientation task), and “the face looked more like that of person A” (face identity task) when the flash appeared; darker pixels indicate the opposite.

Fig. 2a–c show plots of the estimated time windows relative to visual probe (flash) onset in the bar orientation, face orientation, and face identity tasks, respectively. The observers made their decisions based on images presented within a certain frame range around flash onset. The full widths at half-maximum (FWHMs) of the estimated time windows for the bar orientation, face orientation, and face identity tasks were 245.4, 263.4, and 283.1 ms, respectively. Such fairly broad values were anticipated, because each image was 100 ms in duration (this was inevitable, given the need for the visibility of the RSVP), thus artificially inflating time-window estimates by up to 90 ms both forwards and backwards. The time window peak for the bar orientation task occurred 42.9 ms after the flash, indicating that the flash was perceived to lag the changing orientation, consistent with the findings of classical FLE studies. On the other hand, the peak for the face orientation task (Fig. 2b) was at –13.5 ms (i.e., 13.5 ms *before* the flash). Finally, the peak for the face identity task (Fig. 2c) was at –83.3 ms relative to the flash, thus exhibiting a large “flash-lead” effect. The difference from the bar orientation results was thus over 125 ms.

Figure 2d–f are weight maps in the time-intensity domain. The observers made decisions relying not only on the images that differed most radically from the midpoints, i.e., the most obvious and easiest images to report, but also using more-or-less wide ranges of stimulus intensity; the peak timing was relatively constant across different stimulus intensities, thus stimulus discriminability in each task had a negligible effect on the peak timing.

### Auditory probe

**Figure 3:**
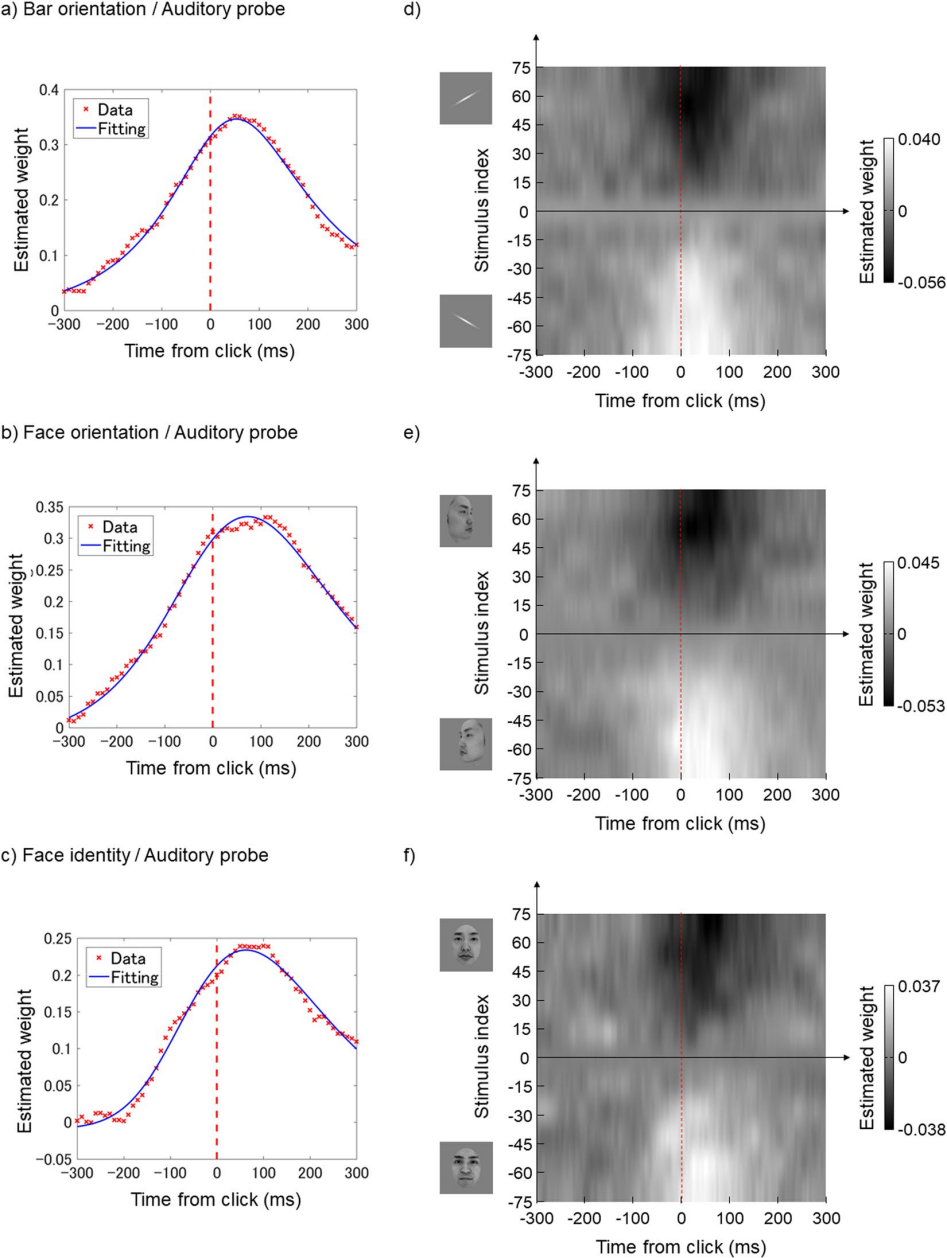
The results of Experiment 1 (click stimulus / auditory probe). Conventions are identical to those in Fig. 2.

Fig. 3a–c show the estimated time windows relative to click onset for the bar orientation, face orientation, and face identity tasks, respectively. Unlike what was seen with the flash, the peak latencies were located after the click in all three tasks (47.8, 82.2, and 78.8 ms), and the variability across tasks was much smaller. The FWHMs of the time windows were 338.0, 378.1, and 345.2 ms for the bar orientation, face orientation, and face identity tasks, respectively, thus wider than those under the flash condition.

Figure 3d–f show weight maps in the time-intensity domain for the bar orientation, face orientation, and face identity tasks, respectively. Evidently, observers made their decisions based on a wide range of stimulus intensities at times after click onset.

**Figure 4:**
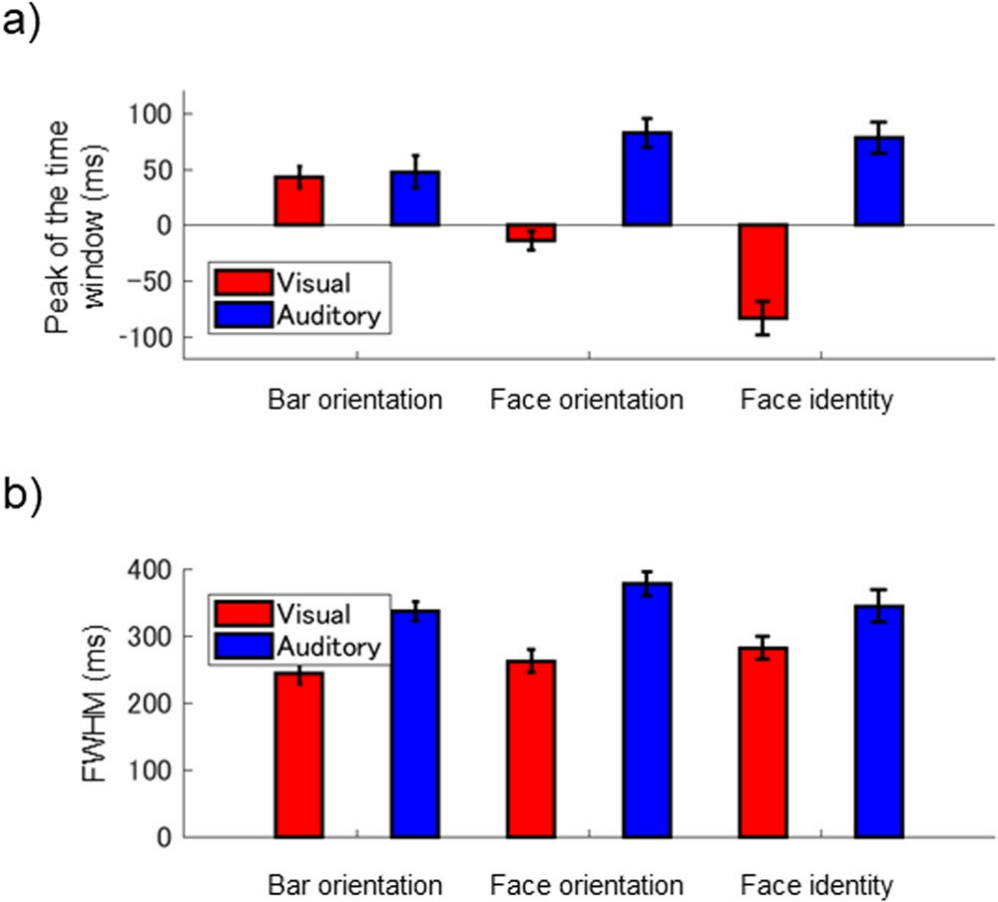
Summary of Experiment 1. The red and blue bars indicate the flash and click conditions, respectively. Error bars indicate ± 1 SE. (**a**) Peaks of estimated time windows averaged across all observers. (**b**) FWHMs of estimated time windows averaged across all observers.

Fig. 4 summarizes the results of Experiment 1; the peaks and FWHMs of the best-fit time windows are plotted in a and b, respectively, for each condition. A two-way analysis of variance (ANOVA) (three visual attributes × two probe modalities) of the peaks revealed significant main effects of visual attribute [F(2, 90) = 7.84, p < 0.01] and probe modality [F(1, 90) = 72.6, p < 0.01], and their interaction [F(2, 90) = 19.66, p < 0.01]. A multiple comparisons test (Tukey’s SDM test, p < 0.05) confirmed significant peak differences among the three tasks for the flash, but not for the click. A two-way ANOVA of FWHM revealed a significant main effect only of probe modality [F(1, 90) = 36.69, p < 0.01]; the time window for a click was significantly broader than that for a flash. The mean peak significantly differed from zero (i.e., flash onset time) in the bar orientation task (t-test, t = 4.50, p < 0.01) and in the face identity task (t = −5.63, p < 0.01) but not in the face orientation task, indicating a “flash-lag” effect in bar orientation and a “flash-lead” effect in face identity when using the visual probe. The peak significantly differed from the click onset time in all three tasks (t = 3.37, 6.39, and 5.64, respectively, p < 0.01) showing “click-lag” effects when using the auditory probe regardless of visual attribute to judge.

## Experiment 2

Variations in time window peaks were revealed above. However, the time window estimated in the RSVP paradigm may not be related directly to the perception associated with a conventional FLE paradigm wherein a visual attribute continuously changes. Here, we used a continuously changing stimulus, and abruptly flipped the direction of change to test whether the “perceived flip time” lag or lead the “physical flip time” depending on tasks and conditions. We determined the perceived bar orientation, face orientation, and face identity at the onset of a probe presented at various times around the flip time to estimate the trajectory of percept relative to the stimulus trajectory (see Figs 1b and S5 in Supplementary Information). We examined whether the observed lag or lead effect was consistent with the observed peak variability in the RSVP paradigm.

## Methods

### Procedure

In the current study, it was extremely difficult for our observers to directly report perceived face orientation or face identity at probe onset. Instead, we determined the subjective neutral points of bar orientation, face orientation, and face identity at the subjective probe onset—in the case of the bar orientation task, the degree of physical inclination of the stimulus required to achieve a subjective appearance of being upright. The trajectory of percept was reconstructed from a set of subjective neutral points measured at several probe onset times (Fig. 1b, see Supplementary Information for details).

The procedure was the same as that of Experiment 1, except that the visual attribute changed continuously. The stimulus index changed at every frame (10 ms). The direction of stimulus change was flipped at certain times during presentation. We used two kinds of sequences: for the CW/CCW sequence, the index was initially set low, increased by one every frame and then after the flip decreased by one every frame; for the CCW/CW sequence, the index was initially set high and then decreased by one every frame and, after the flip, increased by one every frame. The probe appeared at a time randomly chosen from 12 times relative to the flip. In each trial, the total stimulus duration was chosen from the range 700–1100 ms and the probe appeared at a time chosen from the range 350–550 ms after stimulus onset.

In each task, trials evolved according to the staircase method (see Supplementary Information for details); each staircase was designed to converge on the subjective neutral point, i.e., the image *actually* presented at probe onset when the image *seen* at probe onset was neutral (a vertical bar, a front-facing face, or a neutral face). Each observer completed at least two sessions for each task under each probe condition (flash or click). Under the flash condition, N = 11, 10, and 7 for the bar orientation, face orientation, and face identity tasks, respectively; under the click condition, N = 7, 7, and 6, respectively. The mean deviation of the subjective neutral point from the physically neutral stimulus at probe onset was obtained by averaging the stimulus indices between the last two staircase reversals and between the two staircase sequences (with data for the CW/CCW sequence sign-inverted and merged with those for the CCW/CW sequence).

### Data analysis

If one could keep track of what was perceived at each time, then it would be possible to determine whether a flash-lag or flash-lead effect was present by calculating the time shift between the trajectory of the stimulus index, $g(t)$, where *t* = 0 at the flip, and the trajectory of the percept as expressed by the index of the corresponding stimulus, $f(t)$ (for the method of estimating $f(t)$ from the measured deviation of the subjective neutral point, see Supplementary Information); the time lag, ${t}_{d}$, between the two functions was determined by the argument of the maximum of the cross-correlation:

$${t}_{d}={{\rm{argmax}}}_{t}(\int f(\tau )g(t+\tau )d\tau )$$

## Results

**Figure 5:**
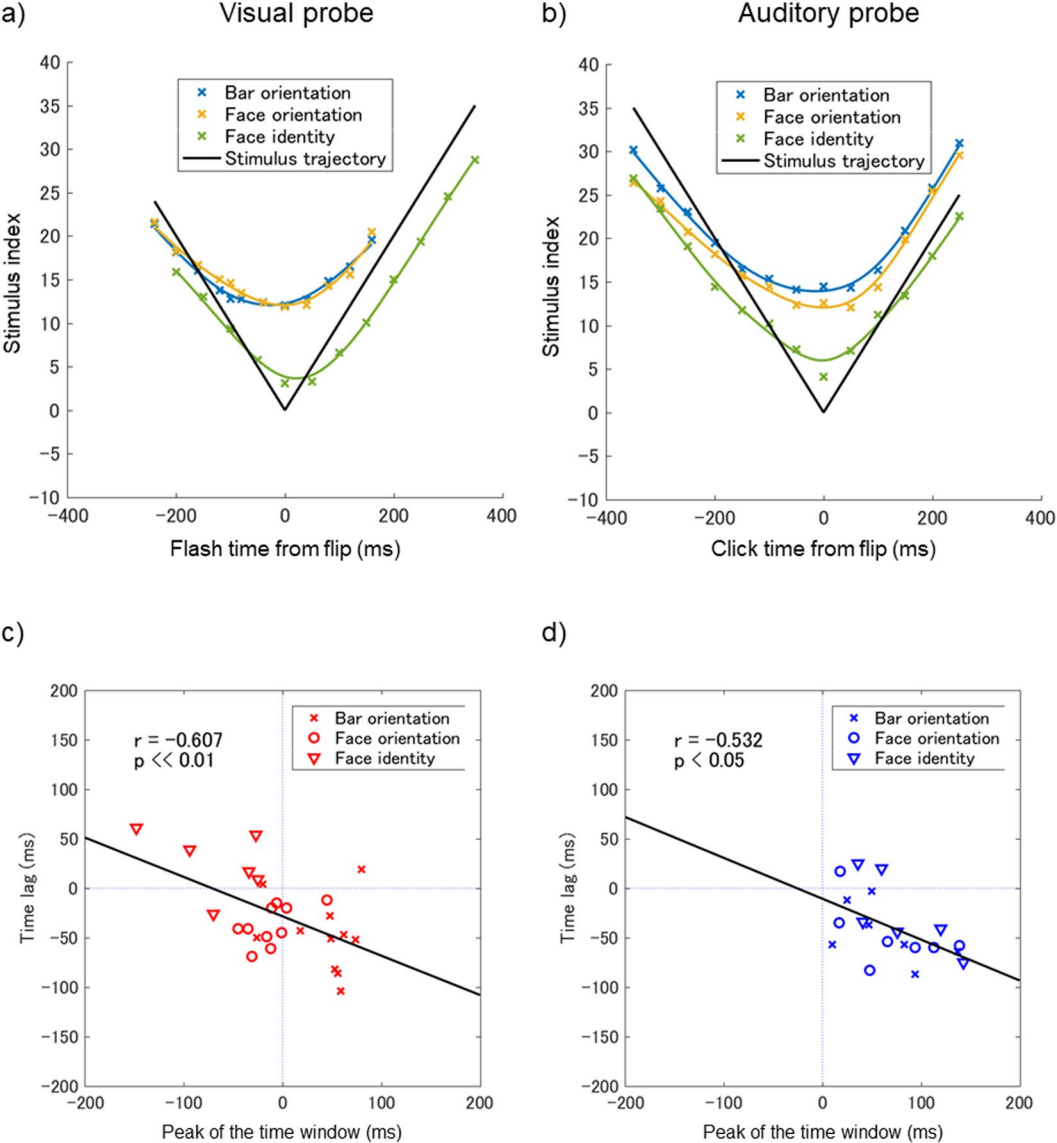
(**a**) Inter-observer average of the estimated trajectory of the percept as a function of visual probe (flash) onset time relative to flip time. Crosses indicate estimates based on data; the solid curves ($f(t)$) are the best-fit cubic spline functions. The V-shaped solid black curve ($g(t)$) indicates the stimulus trajectory. Blue: bar orientation task; Yellow: face orientation task; Green: face identity task. (**b**) Results for the auditory probe (click). (**c**) Scatter plots of all flash conditions and all observer data showing the correlation between the peaks of the time windows in Experiment 1 and the time lags in Experiment 2. The black line is the linear regression line. (**d**) Results for the click.

The upper row of Fig. 5 shows the estimated percept as a function of probe onset time for the bar orientation, face orientation, and face identity tasks. Panels a) and b) show the results under the flash and click conditions, respectively. Under the flash condition, the troughs in the estimated trajectory of the percept were at −31.4 ± 13.0 ms, −1 ± 6.4 ms, and 27.4 ± 10.2 ms for the bar orientation, face orientation, and face identity tasks, respectively. Under the click condition, the troughs were at 5.4 ± 17.7 ms, 2.8 ± 15.4 ms, and −2.8 ± 10.3 ms, respectively. A two-way ANOVA (three visual attributes × two probe modalities) of trough time revealed a significant interaction only [F(2, 41) = 3.42, p < 0.05]. Accordingly, we performed a one-way ANOVA (three visual attributes) of trough time only under the flash condition and confirmed a significant main effect [F(2, 25) = 7.5, p ≪ 0.01]. The estimated trajectory of percept was less sharp than the stimulus trajectory, suggesting that observers made their decisions based on a temporally averaged stimulus over a certain time window (the range estimated in Experiment 1 was 245–380 ms). Unlike in Experiment 1, however, the stimulus in Experiment 2 changed continuously; undesirable confusion caused by stimulus history may have affected the observers’ perception in different ways from task to task, resulting in different apparent vertical shifts in the curves. Below, we focus on the horizontal shift, i.e., the time lag of the estimated trajectory of percept (Fig. S5c and d) and the dependency thereof on visual attributes and probe modality. Under the flash condition, the trajectory of percept in the bar orientation task shifted to the past (i.e., leftward on the plot) compared to the stimulus trajectory. Therefore, the observers tended to make judgments based on images presented after the flash. In the face orientation task, the curve similarly shifted to the past, but somewhat less. Although the results of Experiment 1 show that the peak in the time window in the face orientation task was located slightly before the flash onset (−13.5 ms), the peak time was not significantly different from zero and the time window per se had a slightly longer tail after the flash. This property of the time window in the face orientation task may explain the apparent absence of the flash-lead effect observed in Experiment 2. In the face identity task, the curve shifted slightly to the future, indicating that the observers tended to make decisions based on the images presented before the flash. On the other hand, the three click curves exhibited less marked differences in time shift.

In summary, the estimated trajectories of percept shifted along the time axis compared to the stimulus trajectory, consistent with the flash-lag and flash-lead effects observed in Experiment 1, except for the bias in the vertical axis and the small flash-lag shift in the face orientation task.

The lower row of Fig. 5 shows a scatterplot for all conditions and all observers; the abscissa indicates the peaks of the time windows in Experiment 1 and the ordinate indicates the time lags (${t}_{d}$) in Experiment 2. The correlation was significant (t-test, t = −3.89, p < 0.01 for the flash; t = −2.66, p < 0.05 for the click), indicating that latency differences depending on visual attributes and probe modalities found when using the RSVP paradigm (Experiment 1) were linearly related to the time lags seen in Experiment 2, when continuously changing stimuli were used as in conventional FLE experiments. The slope of the linear relationship was significantly shallower than −1 (t = 5.89, p <<0.01 for the flash; t = 3.79, p ≪ 0.01 for the click), probably because the time lags in Experiment 2 were underestimated upon stimulus flipping. Although the observers were asked to report the percept at probe onset, their decisions could have been compromised by the percept at the flip time, especially when probe onset was close to flip time. Such confusion might also have made the estimated curve broader than it should be in different ways from task to task.

## Experiment 3

If the time window estimated using the RSVP paradigm is a relevant FLE signature, that window should predict how the FLE might depend on the stimuli presented after or before the probe. Thus, we used a continuously changing stimulus without any abrupt flipping, as in typical studies of the FLE (the “complete” condition), and compared the data with those obtained when the same stimulus sequence was presented either within a pre-probe period or within a post-probe period (“probe-terminated” and “probe-initiated” conditions, respectively; see Fig. 1c). If the time window for a certain task deviated sufficiently toward the future after the probe, a judgment under the “complete” condition should be determined principally by the stimulus sequence after the probe. In such a case, judgments made under the “complete” condition should be more similar to those made in the “probe-initiated” condition than in the “probe-terminated” condition. The opposite should be true if the time window deviated sufficiently toward the past relative to the probe.

## Methods

The stimulus display was refreshed every 10 ms and the stimulus index was continuously changed by one unit without any flipping. In the “complete” condition, the index was changed in one direction (either up or down; e.g., CW or CCW bar orientation task); the total duration was randomly chosen from the range 800–1200 ms. The probe onset time was randomly chosen from the range 400–600 ms after stimulus onset. In the “probe-terminated” and “probe-initiated” conditions, the stimulus was presented only before or after the flash, respectively. The observers were instructed to report the content of visual attributes at the termination or the initiation of the stimulus sequence. A blank gray screen was presented during the rest of the period. The FLE for each condition was measured as the subjective neutral point obtained using the staircase method as was done in Experiment 2. Observers who participated in Experiment 2 also performed Experiment 3.

## Results

**Figure 6:**
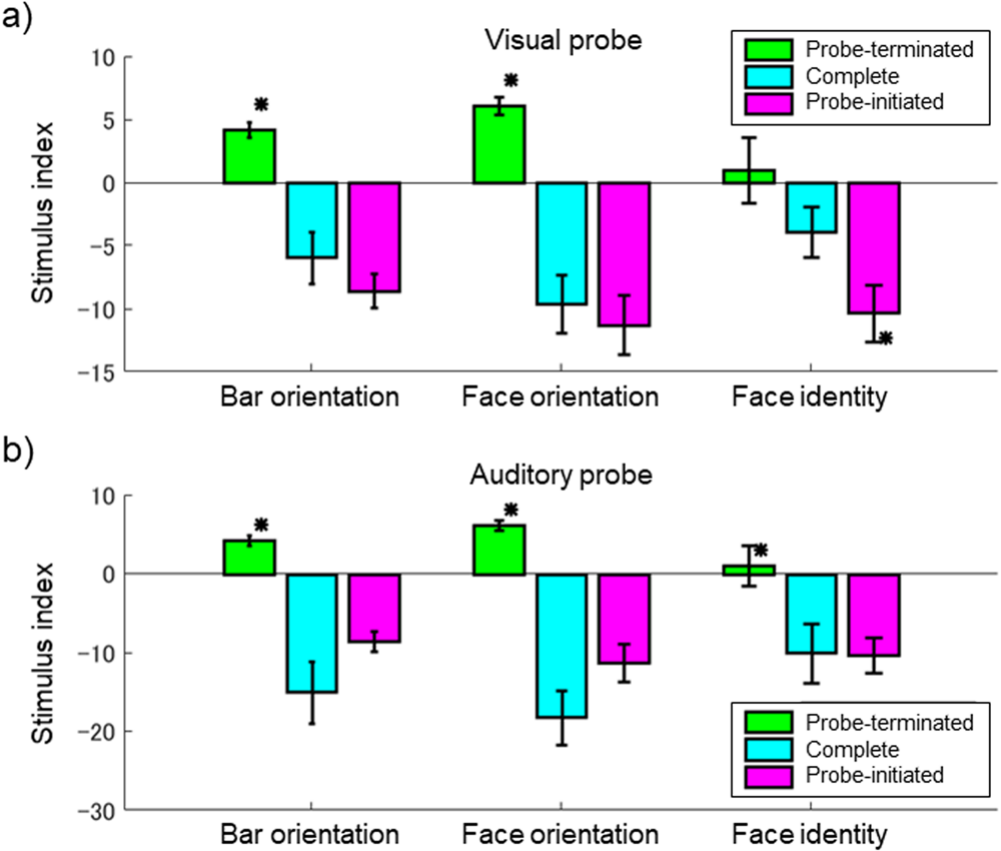
The inter-observer FLE averages for the “probe-terminated” (green), “complete” (cyan), and “probe-initiated” (magenta) conditions. Error bars indicate ± 1 SE. Each asterisk indicates a significant difference (p < 0.05) compared to the value associated with the “complete” condition. (**a**) Results for the flash. (**b**) Results for the click.

The results for the flash are shown in Fig. 6a. In the bar orientation task, the FLE differed significantly between the “probe-terminated” and “complete” conditions (t-test, t = −4.48, p < 0.05) but not between the “complete” and “probe-initiated” conditions. Thus, when observers were asked to report bar orientation at flash onset under the “complete” condition, they relied mainly on images presented after the flash. These results are consistent with those of Experiment 1; the peak in the time window in the bar orientation task was located after the flash and the observers made decisions based primarily on images presented after the flash. In the face orientation task also, the FLE differed significantly between the “probe-terminated” and “complete” conditions (t-test, t = −4.48, p < 0.05) but not between the “complete” and “probe-initiated” conditions. Results of the face orientation task in Experiment 3 were consistent with those in Experiment 2, but apparently not with those in Experiment 1 showing the peak of the time window located slightly before the flash onset. We consider that these results can be reconciled by the longer tail of its time window after the flash than before. Unlike what was seen in the bar orientation and face orientation tasks, the FLE for the face identity task differed significantly between the “complete” and “probe-initiated” conditions (t-test, t = −2.20, p < 0.05) but not between the “probe-terminated” and “complete” conditions. This implies that observers relied on images presented before the flash when judging face identity. These results were consistent with those of Experiment 1, where the peak in the time window in the face identity task was located before the flash.

The click results are shown in Fig. 6b. For all tasks, the FLE differed significantly between the “probe-terminated” and “complete” conditions (t-test, t = −6.02, −8.70, and −2.48 for the bar orientation, face orientation, and face identity tasks, respectively, p < 0.05), but not between the “complete” and “probe-initiated” conditions, consistent with the results of Experiment 1 in which the peak of the time window for all tasks was located after the click.

We found that the results under the “probe-terminated” condition in the bar and face orientation tasks showed statistically significant differences from zero (t-test, t = 6.14 and 8.44, p < 0.01). These results apparently contradict a previous study (Eagleman & Sejnowski^22^) showing that a stimulus similar to that in our flash-terminated condition induced no flash-lag/lead effect when reporting the perceived position of a moving visual stimulus relative to a visual flash’s. In this experiment, we used continuously changing stimuli with visual attributes for which our observers’ performances exhibited a broad “time window” in Experiment 1, and thus stimulus history could impact more on bar- and face-orientation judgement than relative position judgment. It is also noteworthy that the FLE in the flash-terminated condition may depend on several parameters of stimulus configuration such as eccentricity (Kanai *et al*.^23^).

## Discussion

We combined the RSVP with reverse correlation analysis in Experiment 1 and directly estimated weights applied to the visual sequence to characterize the display frames on which observers relied when reporting the contents of visual attributes at probe onset. We found that the peak of the time window varied depending on the visual attribute used and probe modality. In particular, when observers made decisions on face identity at flash onset, a large flash-lead effect was evident. In Experiment 2, we used a continuously changing stimulus with abrupt flips in the direction of change and analyzed time shifts between the trajectories of stimulus and percept. We found that the time shift tended to be predicted by the difference in the peaks of the time windows obtained in Experiment 1. In Experiment 3, we measured FLE as in typical FLE studies to explore whether perception was determined by images presented before or after the probe. Again, the results were explained by the differences in the time windows of Experiment 1.

To date, several hypotheses have been proposed to explain the original FLE of visual motion, and we will particularly focus on two influential models (Krekelberg and Lappe^11^): temporal averaging (Krekelberg & Lappe^24^), and differential latency (Whitney and Murakami^25^; Ogmen *et al*.^26^).

The hypothesis of temporal averaging asserts that the position signals (not light intensity as in Bloch’s law) of a moving stimulus are averaged over a certain period; the FLE occurs because the position of the moving stimulus develops along a trajectory whereas the flash persists at the same position (Krekelberg & Lappe^11^). This theory readily explains the effects of post-flash motion changes on the size of the FLE and the FLE at motion initiation (Eagleman & Sejnowski^22^). However, the interval for averaging estimated in the original study was unrealistically long (approximately 630 ms), probably because of the naïve assumption that only positional signals received after the flash are uniformly averaged.

The hypothesis of differential latency asserts that the FLE occurs because the moving stimulus is of shorter latency than the flash; the moving stimulus has traveled some distance whereas the flash is delayed by several tens of milliseconds, hence appearing to lag. This theory explains the reduction in the size of the FLE when the moving stimulus is dimmed in terms of a reduction in the latency difference (Patel *et al*.^27^), and also explains the perceived trajectory of motion stimulus whose moving direction abruptly flips (Whitney & Murakami^25^). However, the theory does not explain the FLE developing at motion initiation. As a moving stimulus and a flash are physically indistinguishable at onset, a moving stimulus cannot already be of shorter latency, so an additional mechanism such as metacontrast masking and/or attention related to the Fröhlich effect will be required (Fröhlich^28^; Kirshfeld & Kammer^29^; Whitney & Cavanagh^30^. Note that Ogmen *et al*.^26^ offer an integrative explanation of the FLE at motion initiation based on the differential latency hypothesis that takes into account visibility processing as well as position computation.

Studies on the audiovisual FLE are worth mentioning in relation to the controversy over the mechanism of the FLE. Arrighi *et al*. (2005)^20^ reported that the cross-modal FLE of an auditory probe was larger than the FLE of a visual probe. A naïve view of differential latency theory would require that a flash must be processed faster than a tone. However, on the contrary, the neural latency of audition tends to be shorter than that of vision; Arrighi *et al*. regarded such observations as counterevidence for differential latency. However, this is debatable, because the cross-modal FLE may reflect a distinct underlying mechanism; effect size does not necessarily reflect processing latency per se.

The temporal averaging and differential latency theories are not mutually exclusive. A possible explanation reconciling the two theories is to assume that visual information is gathered across a certain time window, but that information is not uniformly averaged over time after the probe arrives, rather being averaged employing a certain “weight” distribution around the time of probe onset (including a period preceding the probe) and the weighting shapes might differ by visual attribute and probe modality. As this hybrid model includes a temporal integration mechanism, the model can naturally explain the FLE evident at motion initiation. In this scheme, the time window can also be viewed as a probabilistic distribution of differential latency.

**Figure 7:**
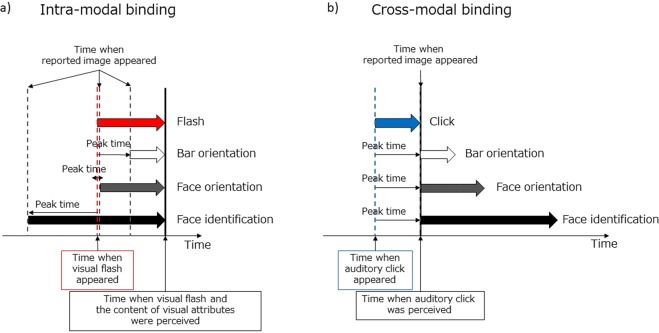
Possible explanations of (**a**) intra-modal binding and (**b**) cross-modal binding. Each bold arrow represents a processing delay in terms of reporting the content of a visual attribute.

The results of Experiment 1 indicate that a certain time window is critical in terms of reporting the content of a visual attribute at probe onset, and imply that the report is generated by integrating stimulus information gathered across a moderate time period, with the peak and interval depending on the visual attribute and probe modality. Experiment 2 showed that the time lag between the percept and stimulus found when the stimulus continuously changed was predictable by the peak shift in Experiment 1, supporting the claim that latency differences among visual attributes elucidated in the RSVP paradigm underlie the FLE. We presume that the peak differences in bar orientation, face orientation, and face identity judgments reflect latency variations among the visual attributes by reference to probe latency (Fig. 7a). Physiological evidence indicates that bar orientation is first encoded in V1 neurons with short latencies (about 35 ms, Lamme and Roelfsema^31^), but that face information is explicitly represented only at the stage of IT, with much longer latencies (109–114 ms for neurons in posterior IT and 123–143 ms for neurons in anterior IT, Schmolesky *et al*.^32^; Lamme & Roelfsema^31^). Psychophysical studies also indicate that reaction times are faster for face detection than for face identification (Barragan-Jason *et al*.^33^); the latter thus requires a longer processing time before it is achieved at a higher stage of the processing hierarchy. All previous studies yielded data consistent with the peak differences revealed in this study.

Notably, the concept of differential latency used here is not limited to neural response delay, rather including any type of processing load that delays awareness of a visual attribute in a broad sense. For example, face identity judgment may require more elaborate inspection than bar orientation judgment, and the sluggishness (or low temporal resolution) of face identification may affect latency in a manner such that observers tend to perceive the last unambiguous image presented before the probe as being the image presented at probe onset.

Rather than calibrating the stimulus range of each task in terms of discriminability, we used the same angular range of stimuli, thus ± 60 deg in the bar and face orientation tasks and morphed face images between two males in the face identity task. Consequently, the observed differences in the peaks of the time windows across tasks might be attributable simply to differences in task difficulty, which could have affected decision making. However, it is unlikely that task difficulty is the only factor explaining the observed differences in the peaks. When we controlled for the difficulty of bar orientation judgment by changing the orientation range, the peaks of the time windows were consistently located after the flash even when bar orientation judgment was as difficult as face orientation or face-identity judgment (data not shown).

When the luminance of a motion stimulus decreases under ordinary FLE conditions, the lag decreases and even becomes a flash-lead effect (Patel *et al*.^27^.). The flash-lead effect reported in the previous study reflects delay in visual signal input to the visual cortex. The flash-lead effect observed in the face-identity judgment in our study is novel in the sense that it is caused by a latency difference attributable to successive hierarchical processing in the visual cortex.

The results of Experiment 1 yield a quantitative estimate of the range of temporal integration. The FWHMs of the estimated time windows were 200–250 ms for the flash condition and 300–350 ms for the click condition, comparable to those of previous studies, implying that perceptual distances between moving and flashed stimuli are based on average positional differences across several hundred milliseconds (Krekelberg & Lappe^24^). The time windows estimated here were, however, distributed both toward the past and into the future, and the peak times exhibited variabilities of several tens of milliseconds depending on the visual attribute, whereas previous studies assumed that visual information was uniformly integrated across an extraordinarily wide range (approximately 500–600 ms) only after probe onset.

Our finding that the FLE was greater for a click than for a flash is consistent with a previous study, although the authors considered their finding as counterevidence for the differential latency hypothesis (Arrighi *et al*.^20^). We also found that the variability in the peaks of the time windows (depending on the visual attribute) became much smaller when a click was used rather than a flash. We thus propose that reporting of visual content at auditory probe onset requires cross-modal binding that is fundamentally different from intra-modal binding.

During intra-modal binding, multiple visual entities are bound to form a unified and coherent visual experience; the content of each visual attribute is updated (with some delay) after every stimulus update. When observers are asked to report the content of a visual attribute at flash onset, the frames of images used for judgment (i.e., the time window) are determined by the differential latency between the probe and the visual attribute of interest (Fig. 7a).

On the contrary, vision and audition are processed separately up to certain stages. During cross-modal binding, the contents of the two modalities represented in separate processing streams are not necessarily bound into a single entity unless accompanied by adaptation, learning, or spatiotemporal congruency. As the click was delivered through headphones, neither a temporal nor a spatial clue was available to the observers to infer click onset relative to the dynamically changing visual stimulus. In such a case, the content of the visual stream experienced before awareness of a click was not recognized as simultaneous with the click. Rather, it is likely that observers began to inspect visual contents after they became aware of the click, interpreting the click as a “go” signal and then reporting the most likely content at the time they heard the click, as if a visual snapshot at the time of the “go” signal were newly registered for this purpose (Fig. 7b). As the processing time needed to become aware of the click was invariant across tasks, the peak of the time window under the click condition was always located after click onset regardless of the visual attribute used. Hine *et al*.^10^ reported a “click-lead” effect when observers were asked to judge whether a click occurred before or after the time when a moving visual stimulus passed the fixation cross. In our framework, this could be accounted for by considering that the time when their observers became aware of the visual event served as the timing of a “go” signal that triggered cross-modal interpretation of the timing of the click. Thus, their results do not contradict our explanation of cross-modal binding.

Postdiction theory (Eagleman and Sejnowski^22^) that involves a mechanism resetting temporal integration and a theory that combines attention, backward masking, and priming (Sheth. Nijhawan, Shimojo^8^) have been proposed as possible accounts for the FLE. Our theory aims to explain the FLE based only on a simple binding mechanism but does not exclude the possibilities that additional, perhaps more higher-order, mechanisms also affect the FLE.

In summary, we estimated the time windows required for bar orientation, face orientation, and face identity judgments, at the time of a flash or click using the RSVP and reverse correlation analysis. We found that the peaks of time windows changed depending on the visual attribute the observers were required to report when the flash occurred. Differential latencies accompanied by temporal integration explained the findings. The results also imply that cross-modal binding differs fundamentally from intra-modal binding in the FLE context.

## Supplementary information

Supplementary Infromation

## Data Availability

Details about our experimental procedure are provided in Supplementary Information.
