# Supplementary material for: Distinct mechanisms of temporal binding in generalized and cross-modal flash-lag effects
Source: Sci Rep. 2019 Mar 7;9:3829. doi: 10.1038/s41598-019-40370-7 (PMC6405767; doi:10.1038/s41598-019-40370-7)
Supplement: Supplementary file 1 — Supplementary Infromation [file 41598_2019_40370_MOESM1_ESM.docx]

Supplementary Information

**Title: Distinct mechanisms of temporal binding in generalized and cross-modal flash-lag effects**

Author: *Ryusuke Hayashi^1^, Ikuya Murakami^2^

Affiliation:

1. Systems Neuroscience Group, Human Informatics Research Institute, AIST, Umezono 1-1-1, Tsukuba, 305-8568, Japan
2. Department of Psychology, The University of Tokyo, 7-3-1 Hongo, Bunkyo-ku, Tokyo, Japan.

**Method**

**Experiment 1**

**Visual Stimuli and Tasks**

In each session, one of the following three tasks was chosen in pseudo-random order (Figure S1):

- **Bar orientation task**: The visual stimuli were white bars, the orientations of which were within ±60 deg from the vertical in 151 steps. We assigned stimulus indices ranging from –75 to +75 to these images, depending on the bar orientation. Thus, bar orientation increased by 60/75 deg as the index increased by one. The task was to report whether the bar seen at the time of the probe tilted counterclockwise (CCW) or clockwise (CW) from vertical. We used 2D Gabor function (sigma of Gaussian envelope in major axis=1.125 deg, sigma in minor axis=0.1125 deg, spatial frequency of cosine modulation in minor axis =0.44 cycle/deg) to generate bar images.
- **Face orientation task**: The visual stimuli were monochromatic facial images in which the head orientation was within ±60 deg in azimuth from the front in 151 steps. As in bar orientation task, we assigned stimulus indices ranging from –75 to +75 to these images, depending on the angle of face orientation. Thus, head orientation increased by 60/75 deg as the index increased by one. The task was to report whether the face seen at the time of the probe faced left or right.
- **Face identity task**: The visual stimuli were monochromatic front facial images morphed from persons A to B (both were Asian males easily distinguished from each other by our observers) in 151 steps. We assigned stimulus indices ranging from –75 to +75 depending on the morphing level. Thus, the face would become more like that of person B by 100/150% of the full morphing range as the index increased by one. The task was to report whether the face seen at the time of the probe looked more like that of person A or that of person B. The same two persons (A and B) were used throughout the study. All facial images were matched in terms of image histograms to minimize difference in low-level features such as luminance and contrast.


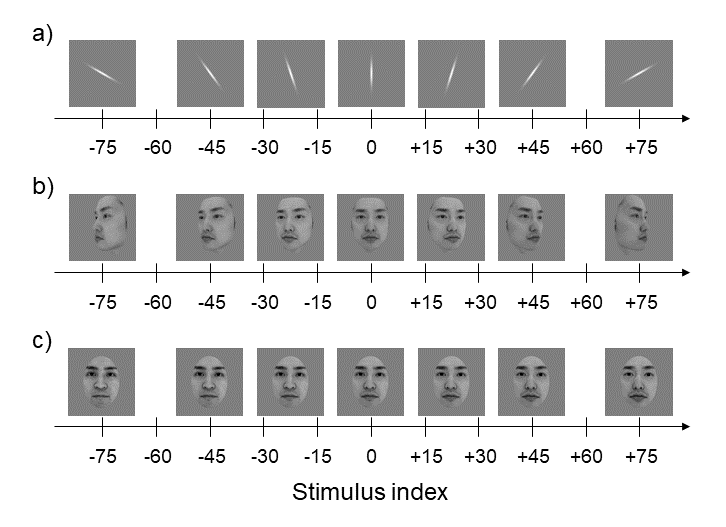


Figure S1: Examples of the images used in a) Bar orientation task, b) Face orientation task, and c) Face identity task. All images were indexed from –75 to +75. The indices “0” in a), b), and c) correspond to a vertical bar, a front-facing face, and a 50/50 morphed neutral face, respectively.

**Estimation of time windows**

We used reverse correlation analysis to estimate the extent to which observers relied on images presented at various times when reporting what they saw at the time of the probe.　To calculate time windows in bar orientation task, the image content at time *t* (in the unit of frame) in each trial, $x\left( t \right)$, was assigned a value of –1, 0, or 1 depending on whether the bar was tilted CCW, vertical, or tilted CW. Responses (*y*) were also assigned a value of –1 or 1 (indicating a “CCW” or “CW” response, respectively). Each temporal window was estimated by calculating $E\left[ y\cdot x(t) \right]$, where *t* = 0 at physical probe onset (Figure S2a). Note that although the images were refreshed every 10 frames, the probe was delivered randomly, with a resolution of only one frame, irrespective of the phase of image duration. Thus, each time window was calculated with a resolution of one frame (10 ms), not 10 frames. In face orientation task, $x(t)$ could be –1, 0, or 1 depending on whether the image at time *t* was a left-facing, front-facing, or right-facing face, and *y* was either –1 or 1 (“left” or “right” response). Similarly, in face identity task, $x(t)$ could be –1, 0, or 1 depending on whether the face was morphed more toward that of person A, neutral, or morphed more toward that of person B; *y* was either –1 or 1 (“person A” or “person B”). Note that in reverse correlation analysis, undesirable confusions, such as priming and aftereffects stemming from the stimulus history, are all canceled out after averaging of data.


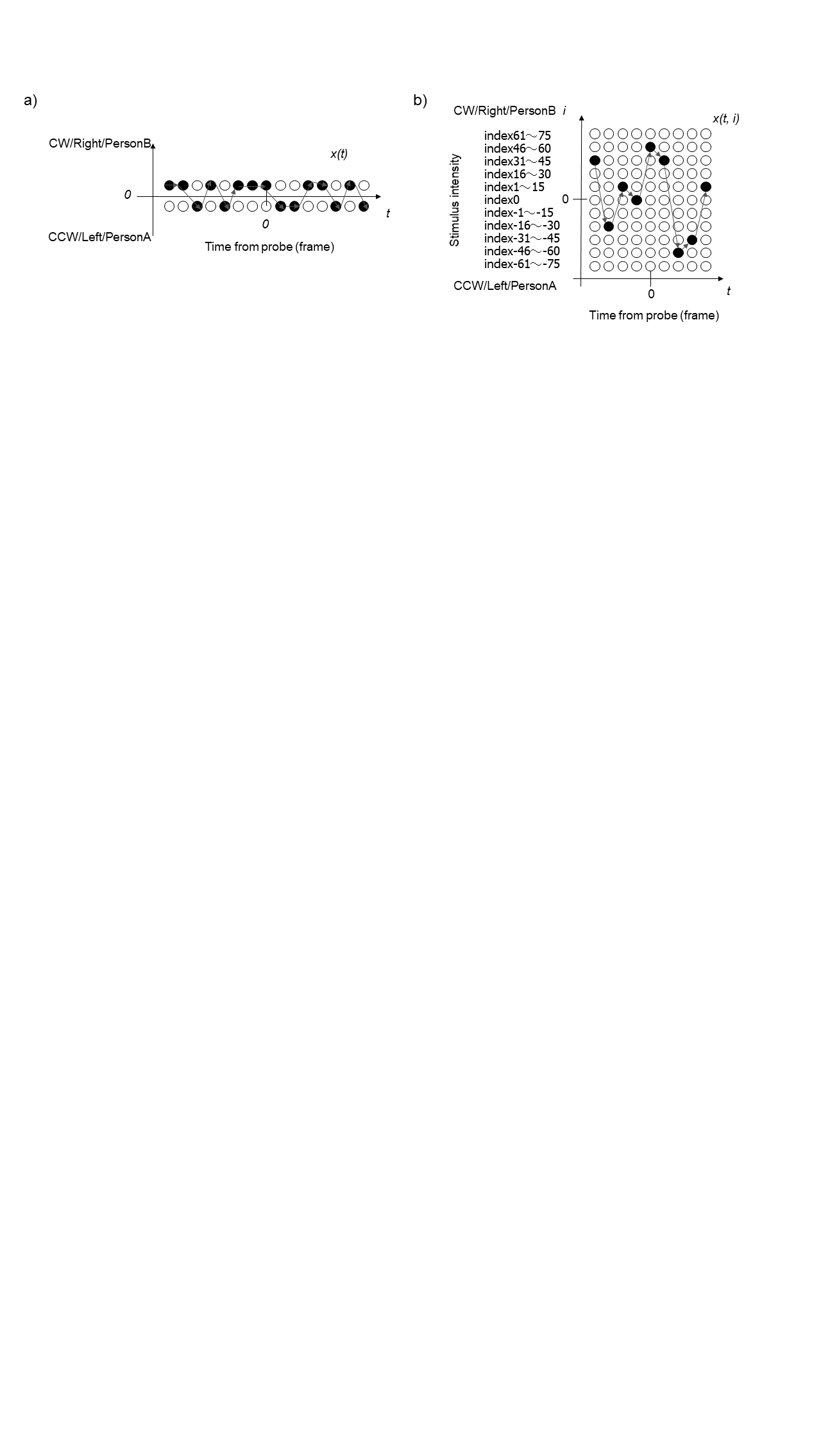


Figure S2: Schematic of the stimulus discretization used when employing reverse correlation analysis to plot a time window (a) and a weight map in the time-intensity domain (b).

It is important to note that the application of reverse correlation analysis in our configuration has several limitations. Reverse correlation analysis requires input as white noise in order to estimate the true shape of the first-order kernel of the system of interest immune to biases from higher-order terms. However, due to the explosion of search space and the necessary number of experimental trials, it is not feasible to sample all possible combinations from stimulus spaces using white noise to characterize a kernel of visual processing, whose stimulus-response function is not simply determined by linear weights of an input image. Instead of using white noise, reverse correlation procedure in psychophysics and neurophysiology frequently relaxes the constraint by using randomly sampled images within a certain range of a focused visual attribute (Murakami, 2001; David et al., 2006). As a trade-off of this compromise, the estimated first-order kernel could be biased by the distribution of input image and possible intrusion of higher-order kernels. Therefore, the estimation of the first-order kernel, or “time window”, in our experiment is valid only within the range of tested stimuli. In Experiment 1, we aimed not to map out which visual element contributed to the observers’ perceptual decision but merely to compare how the impact of each frame around the probe onset made on the decision differed across experimental conditions. We consider that, although there could be biases from higher-order kernels, they should make only limited contributions to the observed differences across experimental conditions. We confirmed that the shape of the first-order kernels did not fundamentally change even if we took into account the second-order kernel (Figures S3 and S4) using a non-linear system analysis method based on Wiener/Volterra series expansion (Hayashi et al., 2017).


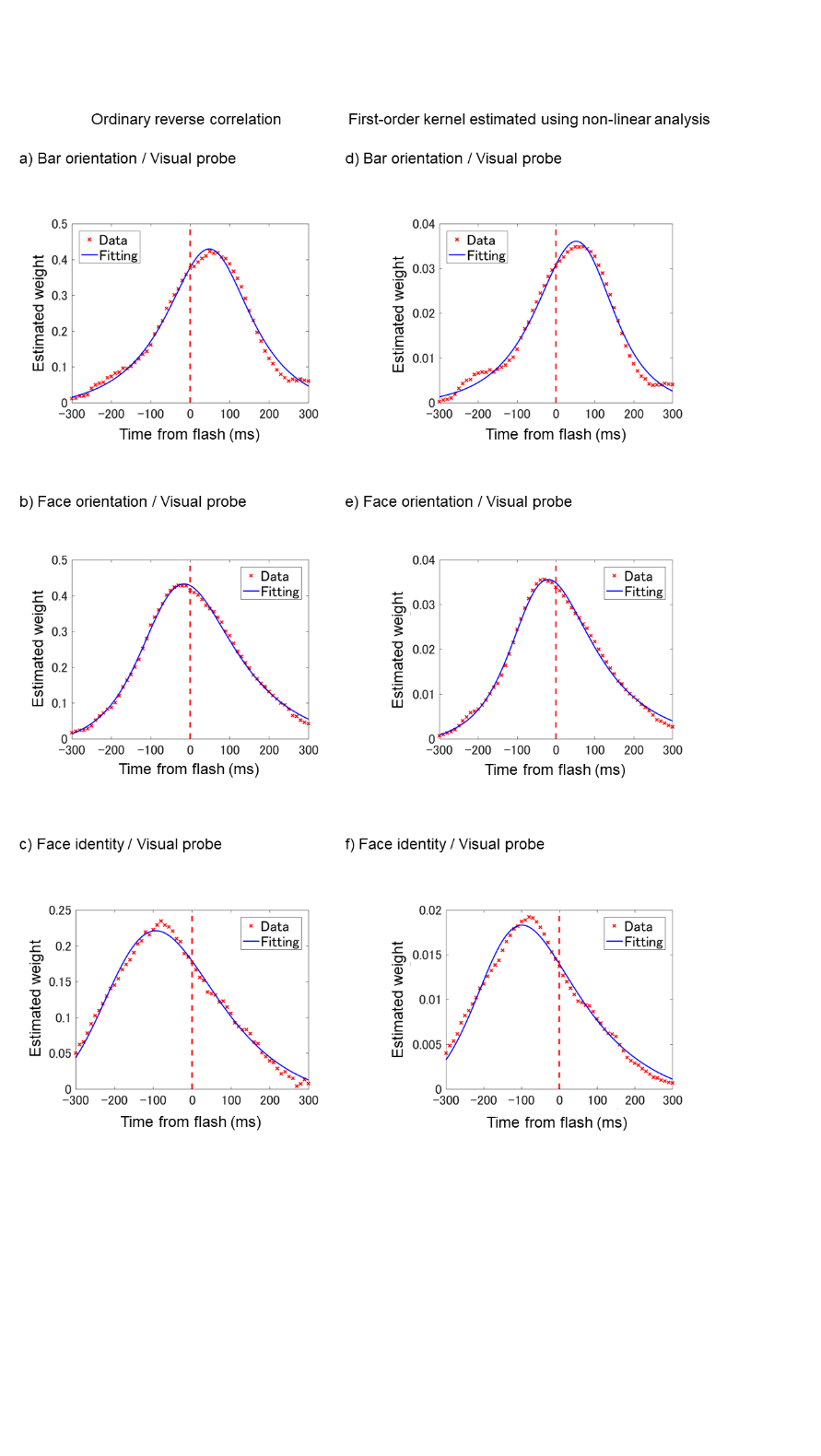


Figure S3: The time windows for the flash, estimated using data across all observers; Left column: the first-order kernels estimated using ordinary reverse correlation analysis. These charts are reproductions of Figure 2a, b, and c in the main text; a) Bar orientation task, b) Face orientation task, and c) Face identity task. Right column: the first-order kernels estimated using non-linear system analysis that took into account the second-order kernels of Wiener/Volterra series expansion; d) Bar orientation task, e) Face orientation task, and f) Face identity task. These results are virtually indistinguishable from each other except for the scales.


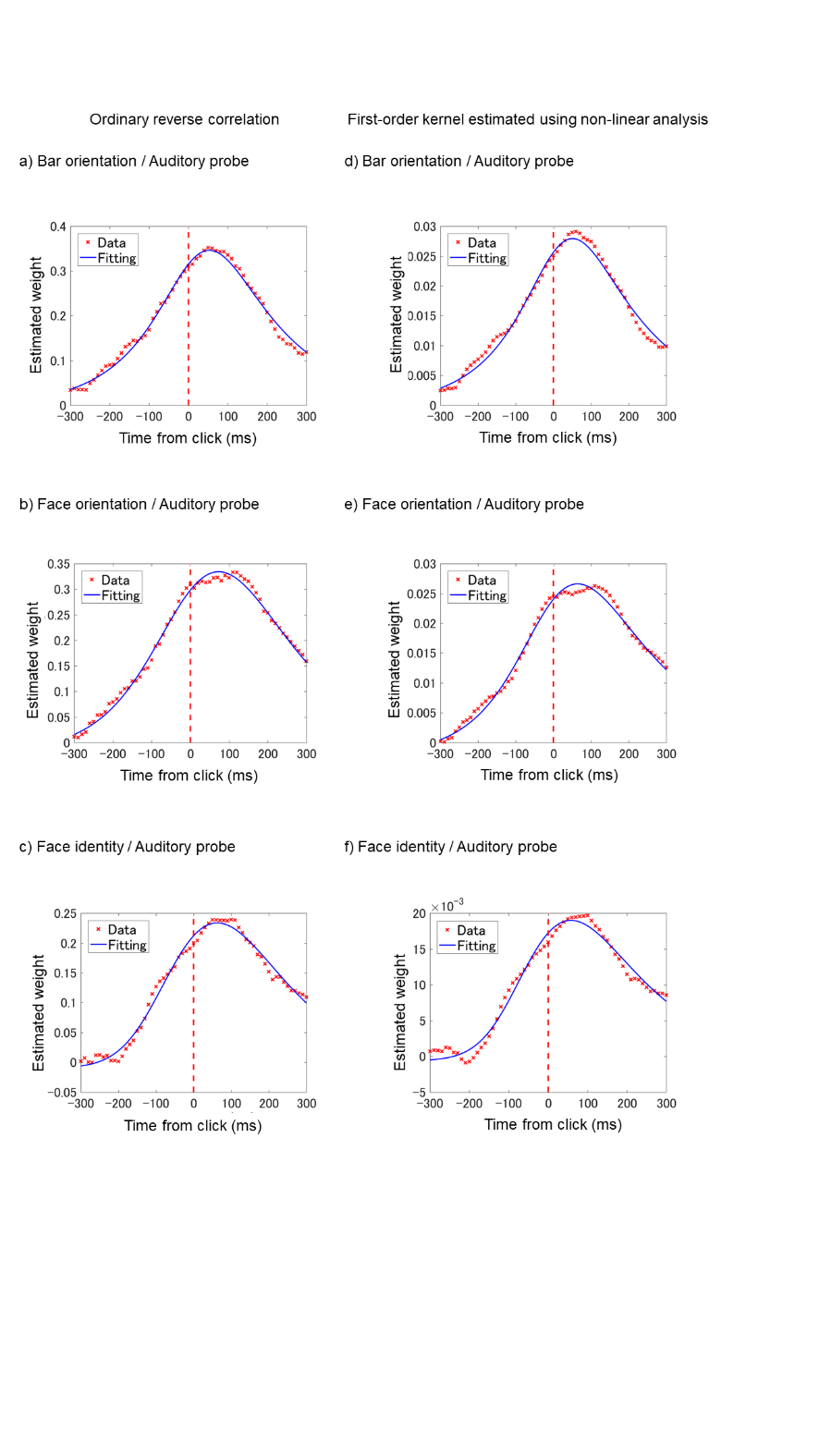


Figure S4: The time windows for the click, estimated using data across all observers; Conventions are identical to those in Figure S3.

**Estimation of weight maps**

We also estimated a two-dimensional window using the joint weights of timing and stimulus intensity in terms of perceptual decision-making. To calculate such a weight map on a plane spanned by time and stimulus intensity, hereafter referred to as the time-intensity domain, the range of stimulus intensity (*i)* was first discretized into 11 bins depending on the index (*i* = 0 when the image was a vertical bar, a front-facing face, and a neutral person in bar orientation, face orientation, and face identity tasks, respectively). Then, each sequential stimulus presentation $x\left( t, i \right)$ for each trial was described as a concatenation of one-hot vectors corresponding to whether image *i* was displayed at time *t* or not (Figure S2b). The weight map was estimated by calculating $E\left[ y\cdot x(t, i) \right]$.

**Experiment 2**

**Experimental procedure**

In a previous study using the same paradigm (Whitney & Murakami, 1998[21]), the effect of a sudden reversal of motion direction on the perceived location of a moving bar relative to a visual flash was directly assessed by measuring the perceived relative locations. However, in the current study it was extremely difficult for our observers to directly report perceived face orientation or face identity at probe onset. Therefore, rather than directly requesting the perceived stimulus content at probe onset, we determined the subjective neutral points of bar orientation, face orientation, and face identity at the subjective probe onset time—in the case of the bar orientation task, the degree of physical inclination of the stimulus required to achieve a subjective appearance of being upright.

The experimental procedure was the same as that of Experiment 1, except that the visual attribute changed continuously. The stimulus index changed at every frame (10 ms). The direction of stimulus change was flipped at certain times during presentation. We used two kinds of sequences: for the CW/CCW sequence, the index was initially set low, increased by one every frame and then after the flip decreased by one every frame; for the CCW/CW sequence, the index was initially set high and then reduced by one every frame and, after the flip, increased by one every frame.

The probe appeared at a time randomly chosen from 12 times relative to the flip. In each trial, the total stimulus duration was chosen from the range 700–1100 ms and the probe appeared at a time chosen from the range 350–550 ms after stimulus onset; the asynchrony between the probe and the flip was chosen from [-240, -200, -160, -120, -100, -80, -40, 0, 40, 80, 120, 160] ms in bar and face orientation tasks and from [-200, -150, -100, -50, 0, 50, 100, 150, 200, 250, 300, 350] ms in face identity task when using visual probe. The asynchrony between the probe and the flip was chosen from [-350, -300, -250, -200, -150, -100, -50, 0, 50, 100, 150, 200, 250] ms in all tasks when using auditory probe.

In each task, the FLE was measured by the staircase method (Figure S5); the staircase was designed to converge on the subjective neutral point, i.e., the image that was *actually* presented at probe onset when the image *seen* at probe onset was neutral (a vertical bar, a front-facing face, or a neutral face). In a two-alternative forced response paradigm, observers were requested to indicate in which way (CW or CCW in bar orientation task, left or right in face orientation task, and person A or person B in face identity task) the image at probe onset appeared inclined. At the beginning of each staircase under each condition, the image that was to be presented at probe onset was set to be physically neutral (zero on the ordinate of Figure S5a). Given the FLE, however, this image would not be the one seen simultaneous with the probe; the image seen simultaneous would be more or less inclined (red dot in Figure S5a). Thus, in the next trial, we made all images throughout the overall sequence slightly less inclined (the upward shift in Figure S5b) to correct for the subjective inclination evident in the first trial.

The staircases for the CW/CCW and CCW/CW sequences were intermingled in the same session. In total, 24 conditions (12 probe timings × 2 sequences) were tested by running 24 staircases in each session for each task. Each staircase was terminated after five staircase reversals. The staircase step size was set to three (in stimulus index units) until the time of the first staircase reversal, and then fixed at one.

Each observer completed at least two sessions for each task under each probe condition (flash or click). Under the flash condition, N = 11, 10, and 7 for bar orientation, face orientation, and face identity tasks, respectively; under the click condition, N = 7, 7, and 6 for bar orientation, face orientation, and face identity tasks, respectively. The mean deviation of the subjective neutral point from the physically neutral stimulus at probe onset was obtained by averaging the stimulus indices between the last two staircase reversals and between the two sequences (with data for the CW/CCW sequence sign inverted merged with those for the CCW/CW sequence).


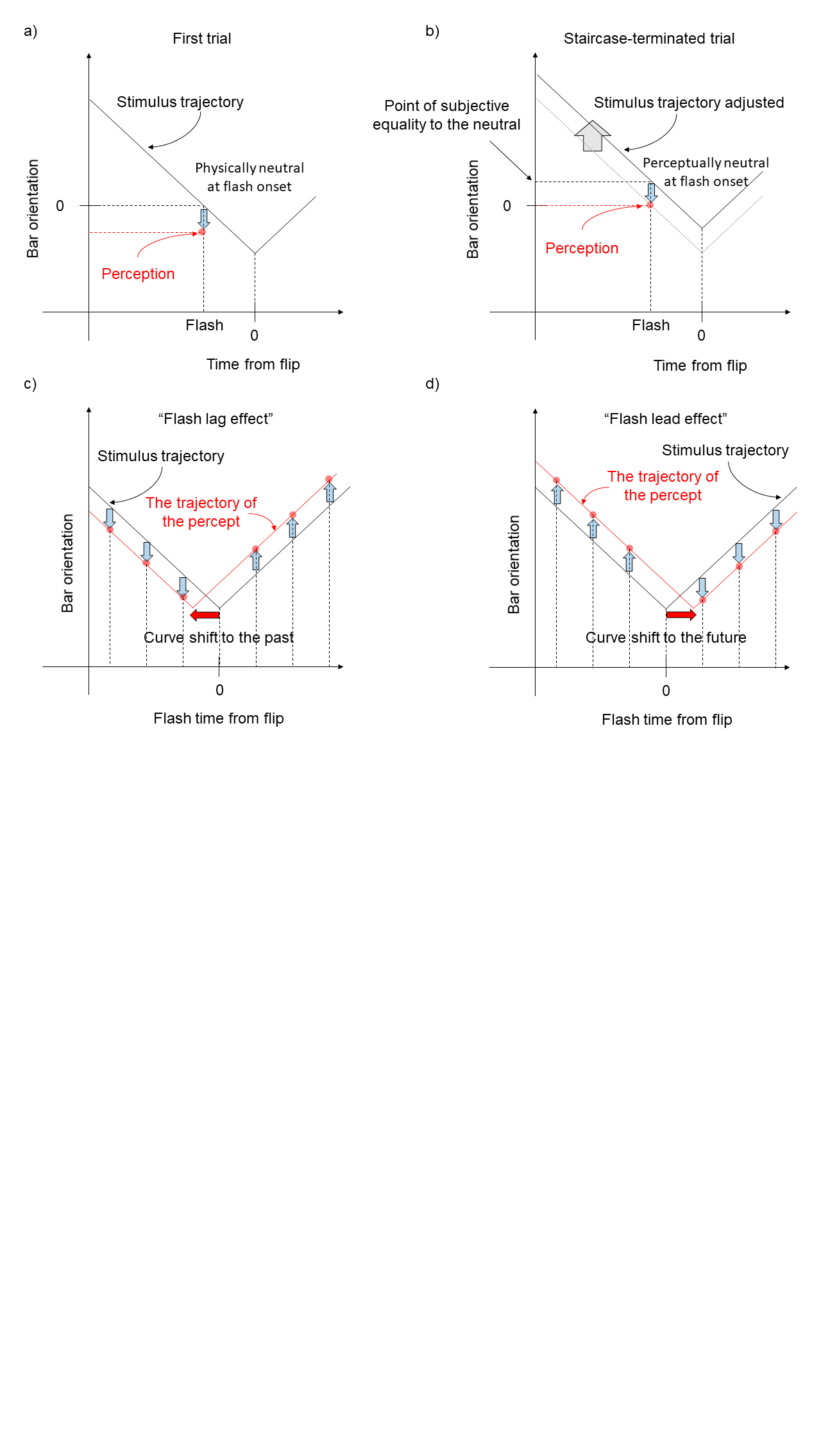


Figure S5: Schematic of Experiment 2 showing how the subjectively neutral point was measured at probe onset. a) Stimulus trajectory in the first trial. Because of the FLE, the image seen simultaneous with the probe was typically biased downward when the stimulus trajectory was downward. b) Stimulus trajectory in the trial in which staircase method was terminated and the subjective neutral point was established. To compensate for the inclination attributable to the FLE, the overall stimulus trajectory was shifted upward, so that a bar with 0 orientation would be seen simultaneous with the probe. The physical deviation from zero at the subjective neutral point was considered as the size of the FLE (blue bold arrow). c) Predicted trajectory of percept when a flash-lag effect occurred. The trajectory of the percept given a certain stimulus trajectory could be estimated by adding the subjective neutral point to the stimulus trajectory at each time point. If the FLE was present, the trajectory of the percept would shift to the past relative to the stimulus trajectory. d) Predicted trajectory of the percept when a flash-lead effect occurred. In this case, the values of the subjective neutral points would be the opposite to the case when the FLE was present and the trajectory of the percept would shift to the future, relative to the stimulus trajectory.

**Cross-correlation analysis to estimate the time lag between the trajectories of the stimulus and the percept**

If one could keep track of what was perceived at each time, then it would be possible to determine whether a flash-lag or flash-lead effect was present by calculating the time shift between the trajectory of the stimulus index, $g\left( t \right)$, where *t* = 0 at the flip, and the trajectory of the percept as expressed by the index of the corresponding stimulus, $f\left( t \right)$. However, what we were able to obtain from the experiment was the subjective neutral point—the degree of physical inclination of the stimulus required to achieve a subjective appearance of being upright—at the subjective probe onset time, and the temporal development thereof ($d\left( t \right)$). We assumed that $d\left( t \right)$depended only on the timing relative to the flip irrespective of the actual stimulus index when the flip occurred. This assumption allowed us to estimate the trajectory of the percept, $f\left( t \right)$, by adding the estimated subjective neutral point, $d(t)$, to $g(t)$, i.e., $f\left( t \right)=d\left( t \right)+g(t)$ (Equation S1; Figures S5c and d, red curves). We focused on the time shift of $f(t)$ relative to $g(t)$ using cross-correlation analysis; the time lag, $t_{d}$, between the two functions was determined by the argument maximum of the cross-correlation:

$t_{d}=\underset{t}{\mathrm{argmax}} \left( \int f\left( \tau\right)g\left( t+\tau\right)d\tau\right)$.
